# Patterns of right ventricular reverse remodeling and hemodynamic drivers in serial balloon pulmonary angioplasty

**DOI:** 10.3389/fmed.2026.1822844

**Published:** 2026-05-20

**Authors:** Lixia Wang, Ziqi Xiong, Qinyan An, Qinhua Zhao, Wenhui Wu, Sugang Gong, Jing He, Hongling Qiu, Cijun Luo, Huiting Li, Jian Xu, Ping Yuan, Rui Zhang, Lan Wang, Hongda Zhang, Jinming Liu, Rong Jiang

**Affiliations:** 1 Tongji University School of Medicine, Shanghai, China; 2 Department of Cardio-Pulmonary Circulation, Shanghai Pulmonary Hospital, Tongji University School of Medicine, Shanghai, China; 3 General Department of Datuan Community Health Service Center of Pudong New Area, Shanghai, China; 4 Department of Radiology, Shanghai Pulmonary Hospital, Tongji University School of Medicine, Shanghai, China; 5 Department of Respiratory, Sijing Hospital of Songjiang District, Shanghai, China; 6 State Key Laboratory of Cardiovascular Disease, Fuwai Hospital, National Center for Cardiovascular Diseases, Chinese Academy of Medical Sciences and Peking Union Medical College, Beijing, China; 7 Department of Cardiology, Shanghai General Hospital, Shanghai Jiao Tong University School of Medicine, Shanghai, China

**Keywords:** balloon pulmonary angioplasty, chronic thromboembolic pulmonary hypertension, echocardiography, pulmonary vascular resistance, right ventricle remodeling

## Abstract

**Background:**

Balloon pulmonary angioplasty (BPA) effectively treats chronic thromboembolic pulmonary hypertension (CTEPH); however, its effects on right ventricular (RV) remodeling remains unclear. This study investigates RV reverse remodeling patterns and key hemodynamic determinants in patients undergoing sequential BPA therapy.

**Methods:**

In this retrospective study, 46 patients who underwent BPA at monthly intervals were included. Serial echocardiography was used to monitor cardiac structure and function, while right heart catheterization assessed hemodynamic parameters before and after treatment. Longitudinal changes were analyzed using mixed-effects models with piecewise assessment of early and late treatment phases.

**Results:**

Significant improvements in right ventricular structure and function were observed across BPA sessions. Notably, right atrial transverse diameter and tricuspid annular systolic velocity demonstrated early responses to BPA, with significant changes evident during the initial treatment phase. In longitudinal analyses, early treatment phases (baseline to BPA_3_) were characterized by marked reverse remodeling, including reductions in right atrial area (RAA), RV end-diastolic transverse diameter, and wall thickness, accompanied by improvements in tricuspid annular plane systolic excursion, enhanced RV function. Greater reductions in pulmonary vascular resistance (PVR) were associated with more pronounced RV reverse remodeling, particularly in RAA.

**Conclusion:**

A temporal pattern of RV reverse remodeling is observed during serial BPA, characterized by substantial early improvements followed by a plateau in later sessions. These changes are closely associated with reductions in PVR, supporting a central role of afterload reduction in driving RV recovery. This pattern may help inform the optimization of BPA treatment strategies.

## 1 Introduction

Chronic thromboembolic pulmonary hypertension (CTEPH), classified as group 4 pulmonary hypertension (PH) in the 2022 European Society of Cardiology/European Respiratory Society (ESC/ERS) guidelines (1), is a recognized sequela of pulmonary embolism. Persistent thrombi evolve into fibrous tissue, obstructing pulmonary arteries and causing PH (2, 3). Pulmonary endarterectomy (PEA) remains the gold standard treatment, providing favorable long-term outcomes and low mortality. However, PEA is not suitable for all patients, particularly those with heavy surgical risk factors or persistent PH post-surgery; pharmacotherapy, such as riociguat, offers an alternative (4).

Balloon pulmonary angioplasty (BPA) has emerged as an effective non-surgical alternative for patients unsuitable for surgery or those with persistent or recurrent PH after PEA. BPA significantly improves hemodynamics, right ventricular (RV) function, exercise capacity, symptoms, and overall clinical outcomes, comparable to PEA (5–8). Although echocardiography and cardiac magnetic resonance imaging reliably assess RV function post-treatment (5, 8–12), detailed mechanisms underlying BPA-induced RV reverse remodeling remain poorly understood.

We hypothesize that BPA can effectively improve RV reverse remodeling in CTEPH patients. Our study aims to: (1) characterize the temporal dynamics of RV reverse remodeling across multiple BPA sessions using echocardiography; (2) examine the alterations in RV-pulmonary artery (PA) coupling and evaluate the impact of RV dilation on left ventricular (LV) structure and function over the course of BPA treatment; and (3) identify the key hemodynamic factors that significantly influence the RV reverse remodeling response to BPA therapy.

## 2 Materials and methods

### 2.1 Design and patients

This retrospective study adhered to the principles stated in the Declaration of Helsinki and was approved by the institutional Ethics Committee (approval number: L25-695). We reviewed the medical records of CTEPH patients who underwent BPA at our institution between May 2020 and May 2024. All enrolled patients met the latest diagnostic criteria for CTEPH, including: a mean pulmonary artery pressure (mPAP) > 20 mmHg, a pulmonary artery wedge pressure (PAWP) ≤ 15 mmHg, and a pulmonary vascular resistance (PVR) > 2 Wood units (WU) at rest, as confirmed by baseline right heart catheterization (RHC) (1). In addition, imaging evidence of thromboembolic obstruction in the pulmonary arteries was required following at least three months of effective anticoagulation therapy (1). Exclusion criteria included patients with incomplete data during treatment, such as missing echocardiographic records.

### 2.2 Data collection

Basic patient information and treatment details were extracted from the medical records. All patients were initially received riociguat, with gradual dose titration, alongside concurrent anticoagulant therapy, to ensure clinical stability over a three-month period. Prior to initiating the BPA treatment protocol, patients underwent the RHC (referred to as pre-RHC) and echocardiography (ECHO_0_).

Following the BPA initiation, the first BPA session (BPA_1_) was followed by subsequent procedures at approximately one-month intervals, labeled sequentially as BPA_2_, BPA_3_, and so on.

The echocardiographic assessment conducted before each BPA session reflects the physiological outcomes of the preceding BPA procedure. Consequently, each inpatient BPA session was labeled as BPA_n_, and its corresponding echocardiographic assessment as ECHO_n-1_.

The operators determined the endpoint of BPA treatment when angiographic assessment confirmed adequate pulmonary artery patency. To evaluate the cumulative hemodynamic effect, a final RHC (post-RHC) was performed three days after the last BPA session.

**Figure 1:**
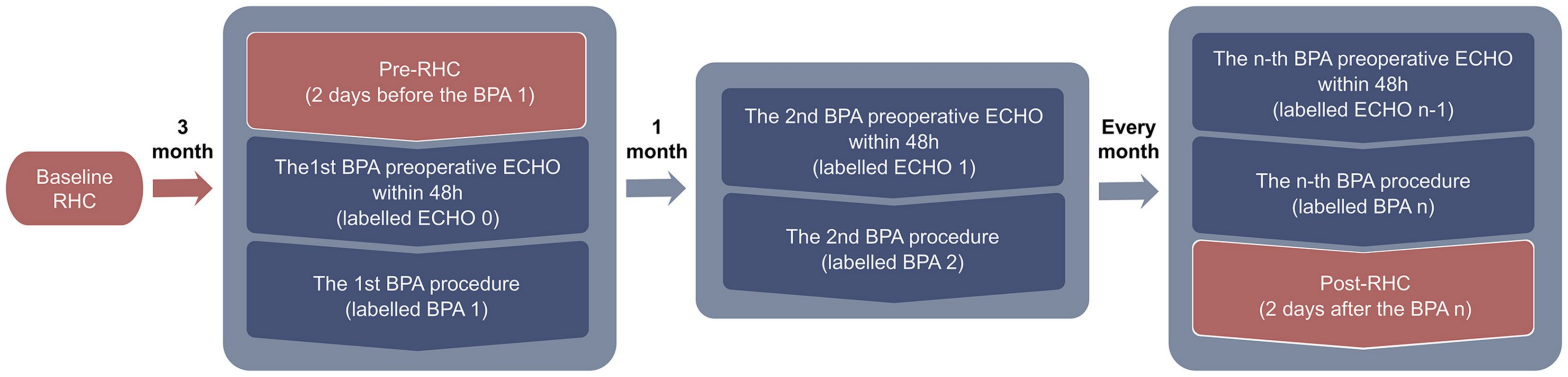
Schematic overview of the BPA treatment process in patients with chronic thromboembolic pulmonary hypertension.

Figure 1 provides an overview of the treatment timeline and the assessments conducted throughout the therapeutic course of these patients. Data from pre-RHC, post-RHC, and all echocardiographic evaluations were retrospectively collected for analysis.

### 2.3 Balloon pulmonary angioplasty

BPA was performed using a standardized procedural framework with individualized, lesion-based decision-making. The number of treated vessels per session was determined by angiographic lesion distribution and patient-specific clinical and hemodynamic status. Procedures were conducted in a staged manner to balance efficacy and safety. The number of treated pulmonary vessels per session was recorded and used as a surrogate of procedural intensity in subsequent analyses. Detailed procedural techniques are provided in the Supplementary material.

### 2.4 Echocardiography

In this study, echocardiographic data were used to evaluate RV reverse remodeling, including right heart structure and function, RV–pulmonary artery (PA) coupling, the impact of RV dilation on LV structure and function, and the pulmonary artery diameter (PAd) during the course of BPA treatment, in accordance with current guideline protocols and reference limits (13, 14). All echocardiographic examinations were performed using the Vivid7 Dimension system (GE Healthcare, USA) by two experienced cardiologists who were blinded to the patients’ clinical history and right heart catheterization (RHC) results.

The right heart structure parameters included the right atrial area (RAA), right ventricular end-diastolic transverse diameter (RVEDTD), right ventricular end-diastolic longitudinal diameter (RVEDLD), right atrial transverse diameter (RATD), right atrial longitudinal diameter (RALD) and right ventricular wall diameter (RVWD). Additionally, RV function was assessed using tricuspid annular plane systolic excursion (TAPSE) and tricuspid annular systolic velocity (TV s’).

In the echocardiographic assessment of hemodynamics, pulmonary artery systolic pressure (PASP) was calculated by summing the tricuspid regurgitation velocity gradient and estimated right atrial pressure (RAP). The TAPSE/PASP ratio served as a key indicator of RV-PA coupling, providing valuable insights into the overall function of the cardiopulmonary system.

Similarly, indices such as left ventricular end-diastolic and end-systolic transverse dimensions (LVEDD and LVESD), along with left ventricular ejection fraction (LVEF), were used to assess LV structure and systolic function. The LV end-diastolic eccentricity index (ENDSEI) served as a key marker reflecting LV compression due to RV enlargement.

### 2.5 Hemodynamic measurement

Hemodynamic parameters assessed by RHC included mPAP, mean right atrial pressure (mRAP), PAWP, cardiac output (CO), cardiac index (CI), and PVR. RHC was performed as previously published in detail (14, 15).

### 2.6 Statistical analysis

Continuous variables were presented as either mean ± standard deviation (SD) or median along with the 25th and 75th percentiles, depending on whether the data followed a normal or a skewed distribution. Categorical variables were expressed as percentages.

To compare RHC parameters and echocardiography parameters before and after BPA treatment, a paired t-test was applied to normally distributed data, while the Wilcoxon signed-rank test was used for non-normally distributed data. Analysis of variance (ANOVA) with Bonferroni correction was utilized to compare the alterations in echocardiography across BPA sessions.

Hemodynamic changes, including ΔmPAP%, ΔPVR%, ΔCI%, and ΔSvO_2_%, were calculated using the formula: (post-RHC – pre-RHC) / pre-RHC × 100%. These changes were then categorized into binary groups based on their median or mean values to classify patients as significant responders (SR) or nonsignificant responders (NSR) to the hemodynamic improvements. To assess trends in echocardiographic changes across consecutive BPA procedures, we employed the repeated measures ANOVA.

To further evaluate the robustness of longitudinal changes across BPA sessions, we applied linear mixed-effects models (LMM) and generalized estimating equations (GEE), which account for unbalanced repeated measures. A random intercept was included in the LMM to account for within-subject correlation. A piecewise time effect was specified to model potential non-linear changes across sessions. In addition, a sensitivity analysis restricted to patients who completed ≥4 BPA sessions was performed to assess the potential impact of changing patient composition across follow-up.

Statistical analyses were performed using SPSS version 25.0 software (SPSS Inc., Chicago, IL, USA) and GraphPad Prism version 9.41 software (GraphPad Software Inc., San Diego, California, USA). A two-tailed *p* value < 0.05 was considered statistically significant.

## 3 Results

### 3.1 Population

A total of 46 CTEPH patients were enrolled in this study, including 12 males and 34 females, with a mean age of 62.1 ± 8.9 years. The distribution of BPA procedures among the patients varied: 4 patients underwent two procedures, 13 patients had three procedures, 13 patients had four procedures, and 16 patients completed five procedures. No statistically significant differences (all *p* > 0.05) in baseline clinical, hemodynamic characteristics were observed across the four groups (Supplementary Table S1). A detailed summary of treated vessel counts across sessions is provided in the Supplementary Materials (Supplementary Table S2).

### 3.2 The effect of BPA treatment on hemodynamics

The hemodynamic data from pre-RHC and post-RHC assessments are summarized in Table 1, demonstrating significant improvements in key parameters such as mPAP, CO, and PVR. Among the 46 patients who underwent BPA treatment, the majority of patients (97.8%) exhibited notable decreases in mPAP and PVR, indicating improved pulmonary circulation. Specifically, 30 patients (65.2%) achieved a post-BPA mPAP of less than 25 mmHg, with 17 patients (37.0%) reducing their mPAP to below 20 mmHg. Additionally, 28 patients (60.9%) experienced a reduction in PVR to below 3 WU.

**Table 1:** Hemodynamic and Echocardiographic changes pre- and post-balloon pulmonary angioplasty.

Variable	Pre-BPA *N* = 46	Post-BPA *N* = 46	*P* value
Age (y)	62.1 ± 8.9		
Female, *n* (%)	34 (75.6%)		
Heart rate, bpm	79.7 ± 15.2	71.9 ± 9.6	0.001
Hemodynamics			
RAP, mmHg	3.0 (2.0, 5.0)	2.0 (0.0, 4.0)	0.018
mPAP, mmHg	48.0 (37.0, 55.0)	22.0 (19.0, 28.0)	< 0.001
PAWP, mmHg	7.0 (5.0, 10.0)	7.0 (4.0, 11.0)	0.555
Cardiac output, L/min	4.9 (3.8, 5.6)	5.6 (4.9, 6.4)	0.001
Cardiac index, L/min/m^2^	2.9 ± 0.9	3.4 ± 0.6	0.002
PVR, Wood units	8.3 (6.6, 11.1)	2.8 (2.6, 3.5)	< 0.001
SvO_2_, %	60.8 ± 7.5	67.6 ± 4.5	< 0.001
RV structural reverse remodeling			
RAA, cm^2^	20.8 (15.6, 25.0)	14.0 (12.1, 17.8)	< 0.001
RATD, cm	4.4 (3.8, 5.2)	3.6 (3.3, 4.0)	< 0.001
RALD, cm	5.0 (4.6, 5.9)	4.4 (4.0, 4.8)	< 0.001
RVEDTD, cm	4.1 (3.7, 4.5)	3.5 (3.2, 3.7)	< 0.001
RVEDLD, cm	2.7 (2.5, 2.9)	4.3 (3.6, 4.6)	0.438
RVWD, cm	0.7 (0.6, 0.7)	0.6 (0.5, 0.6)	< 0.001
RV functional reverse remodeling			
TAPSE, cm	1.9 (1.7, 2.1)	2.0 (1.9, 2.3)	0.001
TV s’, cm/s	11.0 (9.4, 12.3)	12.0 (10.0, 14.0)	0.040
LV structure and function			
LVEDD, cm	4.3 (3.7, 4.5)	4.7 (4.5, 5.0)	< 0.001
LVESD, cm	2.4 ± 0.5	2.7 ± 0.4	< 0.001
LVEF, %	76.0 (70.5, 80.5)	75.0 (68.0, 79.0)	0.146
RV-LV interaction			
ENDSEI	1.2 (1.1, 1.3)	1.0 (1.0, 1.1)	< 0.001
RV-PA coupling			
TAPSE/PASP, mm/mmHg	0.3 ± 0.02	0.4 ± 0.02	< 0.001
Hemodynamics			
PASP, mmHg	75.8 ± 27.2	44.8 ± 9.9	< 0.001
PAd, cm	2.9 ± 0.4	2.7 ± 0.4	0.006

Data expressed as Mean ± SD, or median (quartile range).

CO responses varied among the cohort. While twelve patients experienced a reduction in CO, 34 patients exhibited an increase, indicating positive cardiac adaptations in 73.9% of the cohort. Similarly, 33 patients had improved in CI after BPA, with a 71.7% overall improvement rate. Additionally, the majority of patients (84.8%, *n* = 39) exhibited increased SvO_2_ levels, suggesting enhanced oxygenation in the mixed venous blood, a key indicator of improved circulatory function.

### 3.3 The influence of BPA treatment on echocardiographic findings

Following completion of BPA treatment, echocardiography demonstrated significant improvements in RV structure, function, and hemodynamic parameters. Specifically, normalization of right atrial area (RAA < 18 cm^2^) was observed in 76.1% of patients (35/46), while resolution of RV-induced left ventricular compression (ENDSEI decreased to < 1.0) occurred in 54.3% (25/46). Additionally, pulmonary artery systolic pressure (PASP) decreased to <37 mmHg in 39.1% of patients (18/46).

BPA treatment led to positive effects in RV reverse remodeling. RAA decreased from 20.8 (15.6, 25.0) to 14.0 (12.1, 17.8) cm^2^ (*p* < 0.001), accompanied by marked reductions in both RATD and RALD (*p* < 0.001). There was also a reduction in RVEDTD from 4.1 (3.7, 4.5) to 3.5 (3.2, 3.7) mm (*p* < 0.001), and RVWD decreased from 0.7 (0.6, 0.7) to 0.6 (0.5, 0.6) cm (*p* < 0.001). However, no statistically significant change was observed in RVEDLD following BPA. In terms of RV function, TAPSE increased from 1.9 (1.7, 2.1) to 2.0 (1.9, 2.3) cm (*p* = 0.001), and TV s’ improved from 11.0 (9.4, 12.3) to 12.0 (10.0, 14.0) cm/s (*p* = 0.040), indicating a favorable functional response to BPA.

BPA treatment also has a positive effect on LV remodeling. The significant increase of LVEDD and LVESD (both *p* < 0.001) within the normal range represents the recovery of the LV structure. However, LVEF did not improve after BPA treatment.

BPA treatment resulted in a decrease in PASP from 75.8 ± 27.2 to 44.8 ± 9.9 mmHg (*p* < 0.001), in line with the findings from RHC. TAPSE/PASP and Pad also showed significant improvements after BPA treatment (*p* < 0.001 for both). In addition, the incidence of pericardial effusion was significantly reduced after treatment compared to baseline (*p* = 0.003).

### 3.4 Echocardiographic dynamics during the entire BPA procedure

**Figure 2:**
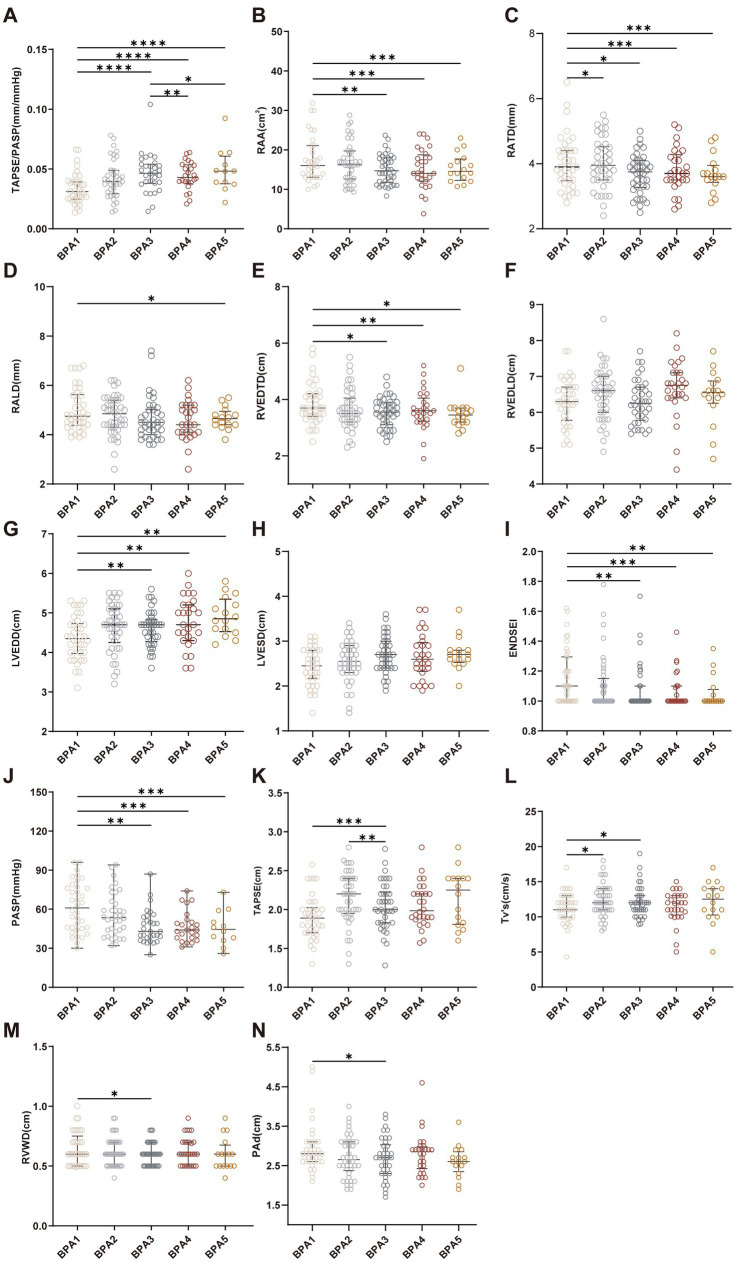
Trends in the echocardiographic parameters throughout a series of balloon pulmonary angioplasty (BPA) procedures. **(A)** TAPSE/PASP; **(B)** right atrial area (RAA); **(C)** right atrial transverse diameter (RATD); **(D)** right atrial longitudinal diameter (RALD); **(E)** right ventricular end-diastolic transverse diameter (RVEDTD); **(F)** right ventricular end-diastolic longitudinal diameter (RVEDLD); **(G)** left ventricular end-diastolic diameter (LVEDD); **(H)** left ventricular end-systolic diameter (LVESD); **(I)** end-systolic eccentricity index (ENDSEI); **(J)** pulmonary artery systolic pressure (PASP); **(K)** tricuspid annular plane systolic excursion (TAPSE); **(L)** tricuspid annular systolic velocity (TV s’); **(M)** right ventricular wall diameter (RVWD); and **(N)** pulmonary artery diameter (PAd). *p < 0.05, **p < 0.01, ***p < 0.001, ****p < 0.0001.

**Figure 3:**
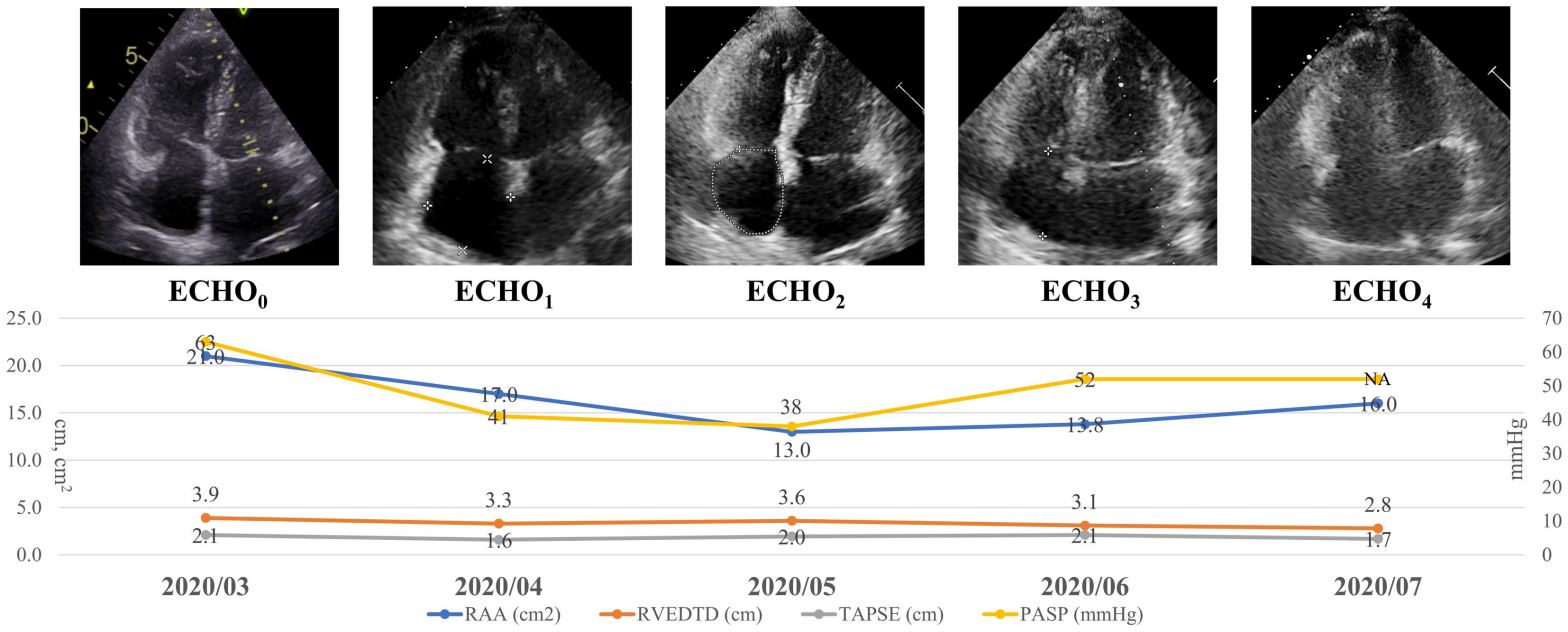
A representative case of a 53-year-old female with chronic thromboembolic pulmonary hypertension (CTEPH) undergoing serial balloon pulmonary angioplasty (BPA). Serial changes in right atrial area (RAA), right ventricular end-diastolic transverse diameter (RVEDTD), tricuspid annular plane systolic excursion (TAPSE), and pulmonary artery systolic pressure (PASP) across BPA sessions are presented. PASP was derived from tricuspid regurgitation (TR) velocity when measurable. At the final time point, PASP could not be reliably estimated due to the absence of an adequate TR signal.

Over the course of treatment, progressive improvements in right heart structure and function were observed (Figure 2 and Supplementary Table S3). Early changes were observed after the initial BPA sessions, with increases in parameters such as RATD and TV s’, suggesting early responsiveness to treatment. As treatment progressed, more comprehensive RV reverse remodeling became evident. Significant reductions were observed in in RAA, RVEDTD, and RVWD, accompanied by improvements in RV systolic function as reflected by TAPSE. Markers of ventricular interaction, including ENDSEI, also showed favorable changes. In addition, improvements were noted in LVEDD, PASP, and RV-PA coupling, assessed by the TAPSE/PASP ratio. A representative case illustrating the improvement in RV structure and function following serial BPA is shown in Figure 3.

Inspection of longitudinal trajectories suggested a pattern of change across BPA sessions, characterized by rapid improvements during the initial sessions followed by a diminished extent of improvement in later sessions. This pattern was consistently observed across multiple echocardiographic parameters.

Based on these observations, a piecewise time effect was applied, separating early (baseline to BPA_3_) and later (BPA_4_–BPA_5_) phases, which was further evaluated using LMM and GEE models. In the piecewise analyses, a consistent pattern was observed across both modeling approaches. During the early phase, most parameters demonstrated significant improvements, including reductions in right atrial and ventricular dimensions, decreases in PASP, and increases in TAPSE (predominantly *p* < 0.001). In contrast, during the late phase, the magnitude of change was markedly attenuated, with most parameters showing non-significant trends (*p* > 0.05), although isolated variables (e.g., RALD) remained statistically significant. A summary of LMM results is presented in Table 2, while the full model outputs for both LMM and GEE are provided in Supplementary Tables S4, S5. In the sensitivity analysis restricted to patients who completed ≥4 BPA sessions, the temporal pattern of early improvement followed by attenuation remained consistent (Supplementary Table S6). The consistency of findings across different statistical approaches supports the robustness of the observed trajectory.

**Table 2:** Summary of longitudinal changes across BPA sessions (LMM analysis).

Parameter	Early phase (baseline to BPA_3_)		Late phase (BPA_4_–BPA_6_)	
	*β*	*P*	*β*	*P*
RAA	−2.03	<0.001	−0.46	0.333
RATD	−0.27	<0.001	−0.10	0.144
RALD	−0.19	0.313	1.05	0.001
RVEDTD	−0.18	<0.001	−0.12	0.063
RVEDLD	0.34	0.443	0.12	0.138
RVWD	−0.02	<0.001	−0.01	0.651
LVEDD	0.16	<0.001	0.07	0.226
LVESD	0.11	<0.001	0.05	0.252
ENDSEI	−0.07	<0.001	−0.01	0.662
PASP	−9.38	<0.001	−2.67	0.279
TAPSE	0.07	<0.001	0.02	0.558
TV’s	0.35	0.015	−0.25	0.332

Early phase: baseline to BPA_3_; Late phase: BPA_4_–BPA_5_. *p* values indicate statistical significance of temporal trends within each phase.

### 3.5 Determinants of RV remodeling changes: hemodynamic factors

We calculated the relative changes (*Δ*%) in key hemodynamic parameters by comparing post- and pre-BPA values. Hemodynamic response stratification was performed by dichotomizing relative changes (Δ%) using cohort median or mean as thresholds. After undergoing BPA treatment, patients with a more substantial reduction in PVR and mPAP achieved better clinical outcomes, characterized as SR.

**Figure 4:**
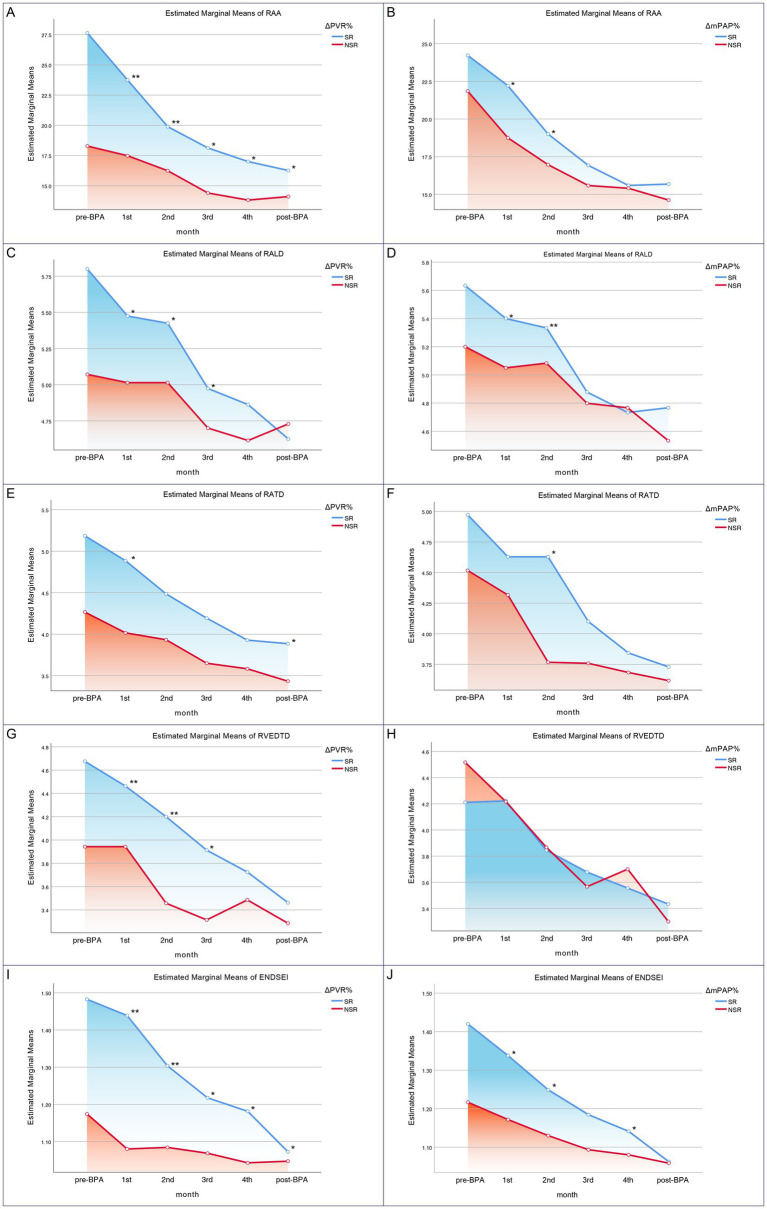
Longitudinal changes in echocardiographic parameters during serial BPA sessions stratified by ΔPVR% and ΔmPAP%. **(A,B)** Right atrial area (RAA); **(C,D)** right atrial longitudinal diameter (RALD); **(E,F)** right atrial transverse diameter (RATD); **(G,H)** right ventricular end-diastolic transverse diameter (RVEDTD); and **(I,J)** end-diastolic eccentricity index (ENDSEI). Panels **A, C, E, G,** and **I** show comparisons stratified by ΔPVR%, while panels **B, D, F, H,** and **J** show comparisons stratified by ΔmPAP%. SR, significant responders; NSR, nonsignificant responders. *p < 0.05, **p < 0.01.

The results showed that ΔmPAP% was −45.6 ± 17.7, ΔPVR% was −63.8 (−74.5, −49.0), ΔCI% was 11.8 (0.03, 55.0), and ΔSvO₂% was 9.4 (2.4, 21.1). Notably, Changes in PVR and mPAP (ΔPVR% and ΔmPAP%) explained the observed trends in RV reverse remodeling, as shown in Figure 4. In contrast, ΔCI% and ΔSvO_2_% inadequately captured the dynamic changes associated with RV remodeling.

Regarding RAA, patients in the ΔPVR%_SR_ group evinced the highest RAA before BPA treatment, indicating more profound RV remodeling. Nevertheless, with each subsequent BPA intervention, the amelioration of RAA was most striking within the ΔPVR%_SR_ cohort, although it was remained higher than in the ΔPVR%_NSR_ group at the end of treatment (Figure 4A). A comparable trend was observed in the ΔmPAP%_SR_ and ΔmPAP%_NSR_ groups (Figure 4B). Consistent trends were noted in the remodeling of right atrial dimensions: both RALD and RATD improved more substantially in the ΔPVR%_SR_ and ΔmPAP%_SR_ groups (Figures 4C–F). Concerning the RVEDTD, patients in the ΔPVR%_SR_ group also had a higher baseline RVEDTD than those in the ΔPVR%_NSR_ group. Successive BPA interventions elicited the most pronounced enhancements in RVEDTD within the ΔPVR%_SR_ group (Figure 4G), but this progressive trend did not emerge in the ΔmPAP%_NSR_ and ΔmPAP%_SR_ groups (Figure 4H).

Patients in the ΔPVR%_SR_ group predominantly presented higher ENDSEI values than the others before BPA treatment, indicating more severe RV compression on the LV. This index underwent the most notable improvements with successive BPA interventions in the ΔPVR%_SR_ group (Figure 4I). A comparable pattern was observed within the ΔmPAP%_SR_ and ΔmPAP%_NSR_ groups (Figure 4J).

In summary, among all assessed hemodynamic parameters, ΔPVR% emerged as the strongest indicator of RV remodeling trends. Of the reverse remodeling indices, RAA provided the most consistent and stable response throughout the treatment course, serving as a reliable marker for tracking structural recovery.

## 4 Discussion

This study suggests a characteristic pattern of RV reverse remodeling across sequential BPA sessions, with pronounced early improvements followed by a diminished extent of improvement in later stages. These changes appear to be closely associated with reductions in pulmonary vascular resistance, supporting a hemodynamic contribution to BPA-related cardiac recovery. These findings provide mechanistic insight into the process of RV adaptation during BPA and may have implications for optimizing treatment strategies in patients with CTEPH.

The ESC/ERS guideline upgrade (IIb-C to I-B) reflects accumulating evidence supporting BPA’s role in inoperable CTEPH (5). Our results align with previous researches (16–20), providing evidence that BPA treatment effectively improves hemodynamics and cardiac systolic function in CTEPH patients.

Notably, our temporal analysis suggests a phased pattern of RV reverse remodeling during sequential BPA sessions. Certain echocardiographic parameters appeared to respond at different stages of the BPA course. RATD showed an early response following the initial BPA session, suggesting its potential as a sensitive early marker, whereas RVEDTD appeared more responsive in reflecting structural remodeling over time. These findings are consistent with previous studies using comprehensive echocardiographic scoring systems to predict severe PH and assess prognosis in patients with chronic lung disease (14, 21). In addition, TAPSE/PASP showed a progressive increase over the treatment course. However, longitudinal analysis of its individual components revealed a dissociation between changes in TAPSE and PASP, suggesting that the observed increase in TAPSE/PASP may be driven predominantly by afterload reduction rather than intrinsic recovery of RV systolic function. Therefore, in the setting of dynamically changing pulmonary pressures, TAPSE/PASP should be interpreted with caution and not considered a standalone marker of RV–PA coupling improvement. Importantly, although improvements in most echocardiographic indices tended to attenuate in later sessions, a plateau rather than a definitive cessation of benefit more appropriately characterizes this trend.

Despite BPA’s demonstrated efficacy, the field currently lacks consensus regarding standardized treatment endpoints. By characterizing the temporal dynamics of RV reverse remodeling and the potential for diminishing returns in later sessions, our findings may help inform the refinement of procedural targets and avoid overtreatment. The observed pattern of RV reverse remodeling should not be interpreted as being driven solely by the number of sessions. Rather, it likely reflects the cumulative treatment effect influenced by procedural efficacy, lesion characteristics, and patient-specific factors. These findings highlight the importance of individualized treatment strategies and may help inform the optimization of procedural targets, rather than relying on a fixed number of sessions alone. Nevertheless, these findings should be interpreted with caution. Given the non-randomized design, potential influences of regression to the mean, survivor bias, and unmeasured confounding cannot be excluded. In addition, although the dataset reflects real-world clinical practice, the generalizability may be constrained by institutional-specific factors including procedural expertise and case complexity.

In this study, the mPAP of some patients did not fall below 20 mmHg after the last BPA treatment, but a significant decrease was seen compared with the pre-RHC results. Therefore, we further explored the different patterns of improvement in RV remodeling in different patients by stratifying the degree of mPAP response, which may provide guidance for personalized treatment strategies. We observed that patients who demonstrated a greater reduction in ΔPVR% and ΔmPAP%, designated “super-responders,” exhibited a greater improvement in RV remodeling. This subgroup analysis emphasizes the influence of hemodynamic response, particularly PVR reduction, on cardiac structural recovery. These findings support the emerging consensus that ΔPVR and ΔmPAP serve as key metrics for evaluating BPA efficacy and safety. Incorporating these metrics into treatment algorithms may facilitate the development of standardized guidelines and promote wider integration of BPA into clinical workflows.

Interestingly, patients with higher baseline RAA often demonstrated larger reductions in PVR and greater RAA improvement. Despite chamber dilation, the right atrium of these patients may retain a degree of structural adaptability, with cardiomyocyte hypertrophy occurring in the absence of intrinsic sarcomeric changes (22). Therefore, once the afterload on the RV is relieved through successful BPA, RV diastolic pressure may fall, leading to reduced RA pressure and subsequent reverse remodeling of the right atrium. Unlike the RV, which undergoes slower and more complex changes, the right atrium—owing to its higher compliance and lower structural inertia—responds more rapidly to hemodynamic unloading. This also explains why RATD was the earliest parameter to show significant improvement following BPA. Patients with higher baseline RAA and better hemodynamic response may be in a dynamic phase of the disease. In contrast, those with smaller right atrial dimensions may experience less hemodynamic burden or represent earlier disease stages, thereby limiting the extent of improvement observed following BPA. Our findings support the notion that RA size not only serves as a marker of disease severity, but may also have prognostic value in identifying patients who stand to benefit most from BPA. However, even with substantial structural improvements, normalized RAA was rarely achieved in patients with advanced remodeling. This underscores the importance of timely intervention. While BPA cannot fully reverse late-stage decompensation, early initiation can maximize therapeutic gains.

This study has certain limitations that should be acknowledged. First, our assessment of RV remodeling relied on conventional echocardiographic evaluation of cardiac structure and function rather than cardiac magnetic resonance imaging. However, given the widespread availability, cost-effectiveness, and practicality of echocardiography, it remains a clinically relevant and useful tool. Second, the relatively small sample size limits the generalizability of our findings. Further studies with larger cohorts are needed to validate current findings across different patient populations. Furthermore, in the absence of a control group receiving medical therapy alone, the independent contribution of BPA cannot be definitively established. Ongoing pharmacological effects during follow-up may also have contributed to the observed improvements, thereby limiting causal inference. Future prospective controlled studies are warranted to disentangle the respective effects of BPA and pharmacotherapy.

## 5 Conclusion

A distinct temporal pattern of RV reverse remodeling is observed during serial BPA, characterized by substantial early improvements followed by a plateau in later sessions. This pattern appears to be closely linked to reductions in PVR, supporting its role as a key hemodynamic driver of RV recovery. These findings may have implications for optimizing BPA treatment strategies. Given the limited and imbalanced sample size, the results should be considered exploratory and require validation in larger cohorts.

## Data Availability

The raw data supporting the conclusions of this article will be made available by the authors, without undue reservation.
