# Supplementary material for: Patterns of right ventricular reverse remodeling and hemodynamic drivers in serial balloon pulmonary angioplasty
Source: Front Med (Lausanne). 2026 May 20;13:1822844. doi: 10.3389/fmed.2026.1822844 (PMC13230164; doi:10.3389/fmed.2026.1822844)
Supplement: Supplementary file 1 [file Data_Sheet_1.docx]

Supplementary Material

# Balloon pulmonary angioplasty

Low-molecular-weight heparin therapy was initiated three days prior to the balloon pulmonary angioplasty (BPA) procedure, replacing oral anticoagulants, and was continued throughout the intervention period. Pulmonary angiography was conducted with emphasis on posteroanterior and left/right anterior (30-degree) projections for accurate identification of the target arteries. BPA was performed in a sequential approach via the right femoral artery. Placement of a 6-F catheter (Johnson & Johnson Vista Brite Tip, Milpitas, California, USA) within the segmental pulmonary artery facilitated access using a 6-F guiding catheter (Medtronic Multi-Purpose, Judkins right 4; Medtronic, Dublin, Ireland) with selective C-arm CT guidance (Siemens Axiom Artis Zee, Erlangen, Germany). After catheter insertion, a nonheparinized heparin solution and a hydrophilic 0.014-inch guidewire (Hi-Torque Pilot 50; Abbott, Santa Clara, USA) were simultaneously introduced into the target artery. Proper placement of an adequately sized semicompliant balloon catheter (Mini Trek; Abbott, Santa Clara, USA) was achieved at the predetermined lesion site. The balloon was inflated with iodinated contrast agent at pressures ranging from 6 to 16 atmospheres for 5 to 20 seconds. If there was no or inadequate response observed in the vessels, a balloon of either consistent or gradually increasing size was employed for further dilation.

# Supplementary Figures and Tables

**Table S1** Baseline characteristics according to total number of BPA sessions

|  | **2**  **(n = 4)** | **3**  **(n = 13)** | **4**  **(n = 13)** | **5**  **(n = 16)** | ***P*** |
| --- | --- | --- | --- | --- | --- |
| **Age (y)** | 62.5 (48.8, 71.0) | 67.0 (59.0, 69.5) | 59.0 (52.5, 67.5) | 65.0 (56.0, 70.0) | 0.526 |
| **Female, n (%)** | 4 (11.8%) | 9 (26.5%) | 9 (26.5%) | 12 (35.3%) | 0.691 |
| **Heart rate, bpm** | 89.0 (65.5, 107.3) | 80.0 (68.5, 88.5) | 76.0 (66.5, 83.5) | 82.0 (65.0, 90.0) | 0.556 |
| **RAP, mmHg** | 2.0 (0.3, 3.8) | 4.0 (1.5, 6.0) | 3.0 (1.5, 5.5) | 2.0 (2.0, 4.0) | 0.589 |
| **mPAP, mmHg** | 36.5 (29.5, 46.5) | 40.0 (31.0, 54.5) | 48.0 (31.5, 62.0) | 49.0 (45.0, 53.0) | 0.300 |
| **PAWP, mmHg** | 9.0 (3.5, 11.5) | 6.0 (5.5, 8.0) | 7.0 (5.0, 10.5) | 7.0 (4.0, 10.0) | 0.632 |
| **Cardiac output, L/min** | 6.0 (4.5, 6.7) | 3.8 (3.1, 5.6) | 4.9 (3.8, 5.3) | 4.9 (4.1, 5.4) | 0.556 |
| **Cardiac index, L/min/m²** | 3.7 (2.7, 4.4) | 2.5 (1.9, 3.4) | 2.7 (2.0, 3.3) | 2.8 (2.5, 3.3) | 0.556 |
| **PVR, Wood units** | 5.2 (3.1, 7.6) | 9.8 (5.4, 12.3) | 8.2 (4.4, 13.5) | 8.8 (8.3, 13.1) | 0.556 |
| **SvO_2_, %** | 62.6 (53.0, 74.4) | 59.8 (49.4, 65.7) | 61.9 (60.3, 65.5) | 60.6 (57.6, 64.2) | 0.470 |

**Table S2** Longitudinal analysis using linear mixed-effects models

| **Session number** | **No. of sessions** | **Treated vessels per session, median (IQR)** |
| --- | --- | --- |
| 1 | 46 | 4 (3, 6) |
| 2 | 46 | 4 (3, 5) |
| 3 | 42 | 5 (4, 6) |
| 4 | 29 | 5 (4, 7) |
| 5 | 16 | 7 (5, 7) |

**Table S3** Comparison of echocardiographic parameters of BPA at different time points

| **Dependent Variable** | **Echo time** | **Echo time** | **Mean Difference** | ***P*** | **Dependent Variable** | **Echo time** | **Echo time** | **Mean Difference** | ***P*** |
| --- | --- | --- | --- | --- | --- | --- | --- | --- | --- |
|  |  |  | [95% CI] |  |  |  |  | [95% CI] |  |
| **RAA** | 0 | 1 | 3.25[-0.34, 6.84] | 0.109 | **RATD** | 0 | 1 | 0.52*[0.04, 1.00] | 0.022 |
|  |  | 2 | 4.09*[0.71, 7.47] | 0.007 |  |  | 2 | 0.54*[0.07, 1.01] | 0.012 |
|  |  | 3 | 5.86*[2.41, 9.31] | <0.001 |  |  | 3 | 0.86*[0.38, 1.34] | <0.001 |
|  |  | 4 | 5.77*[2.00, 9.54] | <0.001 |  |  | 4 | 0.74*[0.21, 1.26] | 0.001 |
|  | 1 | 2 | 0.84[-2.81, 4.49] | 1 |  | 1 | 2 | 0.02[-0.47, 0.51] | 1 |
|  |  | 3 | 2.61[-1.10, 6.32] | 0.469 |  |  | 3 | 0.34[-0.16, 0.84] | 0.57 |
|  |  | 4 | 2.52[-1.48, 6.53] | 0.752 |  |  | 4 | 0.21[-0.33, 0.76] | 1 |
|  | 2 | 3 | 1.77[-1.74, 5.28] | 1 |  | 2 | 3 | 0.32[-0.17, 0.81] | 0.636 |
|  |  | 4 | 1.68[-2.14, 5.50] | 1 |  |  | 4 | 0.20[-0.34, 0.73] | 1 |
|  | 3 | 4 | -0.09[-3.97, 3.79] | 1 |  | 3 | 4 | -0.13[-0.67, 0.42] | 1 |
| **RVEDTD** | 0 | 1 | 0.28[-0.15, 0.71] | 0.65 | **PASP** | 0 | 1 | 12.25[-1.08, 25.58] | 0.098 |
|  |  | 2 | 0.46*[0.05, 0.88] | 0.018 |  |  | 2 | 17.97*[4.64, 31.30] | 0.002 |
|  |  | 3 | 0.58*[0.16, 1.01] | 0.001 |  |  | 3 | 26.83*[12.77, 40.89] | <0.001 |
|  |  | 4 | 0.49*[0.02, 0.96] | 0.033 |  |  | 4 | 26.70*[11.61, 41.78] | <0.001 |
|  | 1 | 2 | 0.19[-0.25, 0.62] | 1 |  | 1 | 2 | 5.72[-8.40, 19.84] | 1 |
|  |  | 3 | 0.31[-0.14, 0.75] | 0.544 |  |  | 3 | 14.58[-0.23, 29.39] | 0.057 |
|  |  | 4 | 0.21[-0.28, 0.70] | 1 |  |  | 4 | 14.44[-1.34, 30.23] | 0.101 |
|  | 2 | 3 | -0.12[-0.32, 0.56] | 1 |  | 2 | 3 | 8.86[-5.95, 23.66] | 0.908 |
|  |  | 4 | 0.03[-0.45, 0.50] | 1 |  |  | 4 | 8.72[-7.06, 24.51] | 1 |
|  | 3 | 4 | -0.10[-0.58, 0.39] | 1 |  | 3 | 4 | -0.13[-16.54, 16.27] | 1 |
| **LVEDD** | 0 | 1 | -0.21[-0.58, 0.17] | 1 | **TAPSE** | 0 | 1 | -0.02[-0.21, 0.17] | 1 |
|  |  | 2 | -0.47*[-0.84, 0.11] | 0.003 |  |  | 2 | -0.26*[-0.45, -0.08] | 0.001 |
|  |  | 3 | -0.46*[-0.83, 0.08] | 0.007 |  |  | 3 | -0.16[-0.35, 0.03] | 0.163 |
|  |  | 4 | -0.52*[-0.93, -0.11] | 0.004 |  |  | 4 | -0.17[-0.38, 0.04] | 0.232 |
|  | 1 | 2 | -0.27[-0.65, 0.12] | 0.506 |  | 1 | 2 | -0.24*[-0.44, -0.05] | 0.006 |
|  |  | 3 | -0.25[-0.64, 0.14] | 0.721 |  |  | 3 | -0.14[-0.34, 0.06] | 0.472 |
|  |  | 4 | -0.32[-0.74, 0.11] | 0.369 |  |  | 4 | -0.15[-0.36, 0.07] | 0.578 |
|  | 2 | 3 | -0.02[-0.37, 0.40] | 1 |  | 2 | 3 | 0.10[-0.10, 0.30] | 1 |
|  |  | 4 | -0.05[-0.47, 0.37] | 1 |  |  | 4 | 0.09[-0.12, 0.31] | 1 |
|  | 3 | 4 | -0.07[-0.49, 0.36] | 1 |  | 3 | 4 | -0.01[-0.22, 0.21] | 1 |
| **ENDSEI** | 0 | 1 | 0.10[-0.03, 0.22] | 0.269 | **TAPSE/PASP** | 0 | 1 | -0.01[-0.01, 0.00] | 0.781 |
|  |  | 2 | 0.14*[0.03, 0.26] | 0.009 |  |  | 2 | -0.01*[-0.02, -0.01] | 0.0001 |
|  |  | 3 | 0.17*[0.05, 0.30] | 0.001 |  |  | 3 | -0.02*[-0.03, -0.01] | <0.0001 |
|  |  | 4 | 0.18*[0.04, 0.32] | 0.003 |  |  | 4 | -0.02*[-0.03, -0.01] | <0.0001 |
|  | 1 | 2 | 0.05[-0.08, 0.17] | 1 |  | 1 | 2 | -0.01[-0.02, 0.00] | 0.106 |
|  |  | 3 | 0.08[-0.05, 0.21] | 0.870 |  |  | 3 | -0.01*[-0.02, 0.00] | 0.002 |
|  |  | 4 | 0.09[-0.06, 0.23] | 0.944 |  |  | 4 | -0.01*[-0.02, 0.00] | 0.039 |
|  | 2 | 3 | 0.03[-0.10, 0.16] | 1 |  | 2 | 3 | 0.00[-0.01, 0.01] | 1 |
|  |  | 4 | 0.04[-0.10, 0.18] | 1 |  |  | 4 | 0.00[-0.01, 0.01] | 1 |
|  | 3 | 4 | 0.01[-0.14, 0.15] | 1 |  | 3 | 4 | 0.00[-0.01, 0.01] | 1 |
| **LVESD** | 0 | 1 | -0.07[-0.36, 0.22] | 1 | **RALD** | 0 | 1 | 0.14[-0.38, 0.66] | 1 |
|  |  | 2 | -0.19[-0.47, 0.09] | 0.602 |  |  | 2 | 0.36[-0.14, 0.86] | 0.43 |
|  |  | 3 | -0.33*[-0.62, -0.04] | 0.014 |  |  | 3 | 0.49[-0.02, 1.01] | 0.072 |
|  |  | 4 | -0.30[-0.61, 0.02] | 0.085 |  |  | 4 | 0.58*[0.01, 1.14] | 0.041 |
|  | 1 | 2 | -0.12[-0.41, 0.18] | 1 |  | 1 | 2 | 0.22[-0.31, 0.75] | 1 |
|  |  | 3 | -0.26[-0.56, 0.04] | 0.163 |  |  | 3 | 0.35[-0.19, 0.89] | 0.645 |
|  |  | 4 | -0.22[-0.55, 0.10] | 0.541 |  |  | 4 | 0.44[-0.15, 1.02] | 0.355 |
|  | 2 | 3 | -0.14[-0.44, 0.15] | 1 |  | 2 | 3 | 0.13[-0.39, 0.66] | 1 |
|  |  | 4 | -0.11[-0.43, 0.21] | 1 |  |  | 4 | 0.22[-0.36, 0.79] | 1 |
|  | 3 | 4 | 0.03[-0.29, 0.36] | 1 |  | 3 | 4 | 0.08[-0.50, 0.69] | 1 |

**Table S4** Linear mixed-effects model (LMM) analysis of longitudinal changes across BPA sessions using piecewise (early vs late phase) modeling

|  | **RAA** | **RATD** | **RALD** | **RVEDTD** | **RVEDLD** | **RVWD** | **LVEDD** | **LVESD** | **ENDSEI** | **PASP** | **TAPSE** | **TV’s** |
| --- | --- | --- | --- | --- | --- | --- | --- | --- | --- | --- | --- | --- |
| Early phase | | | | | | | | | | | | |
| β₁ | -2.028 (-2.536, -1.521) | -0.265 (-0.337, -0.193) | -0.191 (-0.563, 0.181) | -0.178 (-0.249, -0.107) | 0.338 (-0.534, 0.121) | -0.024 (-0.037, -0.010) | 0.160 (0.093, 0.227) | 0.109 (0.061, 0.158) | -0.070 (-0.093, -0.048) | -9.382 (-11.984, -6.781) | 0.073 (0.037, 0.110) | 0.345 (0.068, 0.622) |
| *P_1_* | <0.001 | <0.001 | 0.313 | <0.001 | 0.443 | <0.001 | <0.001 | <0.001 | <0.001 | <0.001 | <0.001 | 0.015 |
| Late phase | | | | | | | | | | | | |
| β_2_ | -0.464 (-1.410, -0.481) | -0.100 (-0.235, 0.035) | 1.053 (0.422, 1.684) | -0.118 (-0.243, 0.007) | 0.124 (-0.041, 0.288) | -0.006 (-0.032, 0.020) | 0.074 (-0.047, 0.195) | 0.053 (-0.038, 0.144) | -0.009 (-0.048, 0.031) | -2.672 (-7.537, 2.194) | 0.020 (-0.047, 0.087) | -0.252 (-0.763, 0.260) |
| *P_2_* | 0.333 | 0.144 | 0.001 | 0.063 | 0.138 | 0.651 | 0.226 | 0.252 | 0.662 | 0.279 | 0.558 | 0.332 |

**Table S5** Generalized estimating equations (GEE) analysis of longitudinal changes across BPA sessions using piecewise (early vs late phase) modeling

|  | **RAA** | **RATD** | **RALD** | **RVEDTD** | **RVEDLD** | **RVWD** | **LVEDD** | **LVESD** | **ENDSEI** | **PASP** | **TAPSE** | **TV’s** |
| --- | --- | --- | --- | --- | --- | --- | --- | --- | --- | --- | --- | --- |
| Early phase | | | | | | | | | | | | |
| β₁ | -2.031 (-2.494, -1.568) | -0.275 (-0.339, -0.211) | -0.391 (-0.781, -0.002) | -0.181 (-0.236, -0.126 ) | 0.028 (-0.044, 0.100) | -0.024 (-0.036, -0.012) | 0.160 (-0.042, 0.165) | 0.107 (0.055, 0.158) | -0.070 (-0.093, -0.048) | -9.427 (-11.723, -7.130) | 0.070 (0.041, 0.098) | 0.321 (0.093, 0.550) |
| *P_1_* | <0.001 | <0.001 | 0.049 | <0.001 | 0.447 | <0.001 | <0.001 | <0.001 | <0.001 | <0.001 | <0.001 | 0.006 |
| Late phase | | | | | | | | | | | | |
| β2 | -0.196 (-0.984, 0.592) | -0.051 (-0.143, 0.041) | 1.583 (-1.306, 4.472) | -0.096 (-0.213, 0.021) | 0.127 (-0.049, 0.304) | -0.002  (-0.029, 0.024) | 0.061 (-0.042, 0.165) | 0.022 (-0.061, 0.105) | -0.003 (-0.023, -0.016) | -1.918 (-4.732, 0.897) | 0.019 (-.042, 0.080) | -0.236 (-0.817, 0.346) |
| *P_2_* | 0.626 | 0.274 | 0.283 | 0.109 | 0.158 | 0.862 | 0.224 | 0.606 | 0.730 | 0.182 | 0.535 | 0.427 |

**Table S6** Sensitivity analysis restricted to patients undergoing ≥4 BPA sessions

|  | **RAA** | **RATD** | **RALD** | **RVEDTD** | **RVEDLD** | **RVWD** | **LVEDD** | **LVESD** | **ENDSEI** | **PASP** | **TAPSE** | **TV’s** |
| --- | --- | --- | --- | --- | --- | --- | --- | --- | --- | --- | --- | --- |
| Early phase | | | | | | | | | | | | |
| β₁ | -2.325 (-2.943, -1.708) | -0.284 (-0.332, -0.194) | -0.214 (-0.738, 0.308) | -0.185 (-0.274, -0.097) | 0.049 (-0.057, 0.154) | -0.019 (-0.037, -0.001) | 0.160 (0.072, 0.248) | 0.118 (0.059, 0.176) | -0.085 (-0.114, -0.057) | -10.465 (-13.737, -7.192) | 0.080 (0.031, 0.129) | 0.419 (0.062, 0.776) |
| *P_1_* | <0.001 | <0.001 | 0.418 | <0.001 | 0.362 | 0.036 | <0.001 | <0.001 | <0.001 | <0.001 | 0.002 | 0.022 |
| Late phase | | | | | | | | | | | | |
| β_2_ | -0.434 (-1.433, 0.564) | -0.101 (-0.245, 0.043) | 1.037 (0.290, 1.783) | -0.136(-0.271, 0.000) | 0.091 (-0.086, 0.268) | -0.015 (-0.045, 0.013) | 0.078 (-0.058, 0.213) | 0.058 (-0.039, 0.155) | -0.008 (-0.051, 0.035) | -3.005 (-8.29, 2.28) | 0.116 (-0.065, 0.088) | -0.325 (-0.907, 0.257) |
| *P_2_* | 0.390 | 0.166 | 0.007 | 0.050 | 0.308 | 0.298 | 0.259 | 0.239 | 0.707 | 0.262 | 0.764 | 0.270 |
